# Frequently used antiemetic agent dexamethasone enhances the metastatic behaviour of select breast cancer cells

**DOI:** 10.1371/journal.pone.0274675

**Published:** 2022-09-15

**Authors:** Martin Crozier, Janice Tubman, Bre-Anne Fifield, Rosa-Maria Ferraiuolo, Jenna Ritchie, Katie Zuccato, Emily Mailloux, Indrajit Sinha, Caroline Hamm, Lisa A. Porter

**Affiliations:** 1 Department of Biomedical Sciences, University of Windsor Ontario, Windsor, ON, Canada; 2 Karmanos Cancer Centre, Wayne State University School of Medicine, Detroit, MI, United States of America; 3 School of Liberal Arts and Sciences, Humber Institute of Technology and Advanced Learning, Toronto, ON, Canada; 4 Acenzia Inc., Tecumseh, ON, Canada; 5 Windsor Regional Hospital, Windsor, ON, Canada; University of Tennessee Health Science Center, UNITED STATES

## Abstract

Glucocorticoids, such as dexamethasone (Dex), are used to prevent common side effects induced by chemotherapy and are heavily prescribed for solid cancers such as breast cancer. There is substantial pre-clinical data to support that Dex activation of the glucocorticoid receptor overrides chemotherapy-induced apoptosis in breast cancer cell lines. These findings are compounded by a recent study demonstrating that increased glucocorticoid receptor activation by endogenous stress hormones increased breast cancer heterogeneity and metastasis. Our study is the first to use both *in vitro* and *in vivo* models to thoroughly compare the Dex response on the migration of multiple estrogen receptor negative (ER-) and ER+ cancer cell lines. ER+ and ER- breast cancer cell lines were studied to compare their endogenous glucocorticoid activity as well as their metastatic ability in response to Dex treatment. We show that in the ER- breast cancer lines, Dex increases cell numbers, invasiveness, and migration, while decreasing apoptotic ability. Furthermore, we show that following Dex treatment, ER- breast cancer lines migrate further in an *in vivo* zebrafish model in comparison to ER+ cell lines. The use of ROR1 antibody to block WNT signaling diminished the metastatic properties of ER- cells, however recombinant WNT5A alone was not sufficient to induce migration. Taken together, we demonstrate that Dex treatment exacerbates the metastatic potential of ER- but not ER+ cells. These findings add to the growing body of data stressing the potential adverse role of endogenous and synthetic glucocorticoids in breast cancer biology.

## Introduction

Breast cancer (BC) is a heterogeneous disease that can be classified into subtypes based on gene expression profiles or histological presentation of levels/presence of hormone or growth factor receptors, e.g., estrogen receptor (ER), progesterone receptor (PR), and epidermal growth factor receptor HER2/neu [1, 2]. These receptors serve as molecular targets for many conventional anti-cancer therapies, e.g., Tamoxifen and Trastuzumab. Approximately 15% of BCs lack these receptors and are denoted, triple negative breast cancer (TNBC). TNBC comprises a younger group of patients and outcomes are statistically less positive, largely due to the lack of directed therapies [3]. Most BC related deaths are due to metastatic spread of the disease and not the primary tumour itself [4]. For disease progression and metastasis to occur, cells must acquire several characteristics including the ability: 1) to survive (elude apoptosis) and grow/proliferate at the primary tumour site; 2) to invade through boundaries at the primary site, and 3) to move or migrate, whether in circulation or within neighboring tissues [5]. TNBC and metastatic ER-positive (ER+) or PR-positive (PR+) disease relies heavily on standard of care chemotherapy [3].

Chemotherapy has saved countless cancer patients’ lives over the past thirty years [6]. Unfortunately, these treatments, and in many cases the vehicle in which they are dissolved, cause unwanted side effects. To lessen and even prevent some of these side effects, synthetic glucocorticoids (GCs), e.g., dexamethasone (Dex), are administered in advance of chemotherapy [7]. Dex mediates anti-emetic effects by inhibiting the glucocorticoid receptor (GR). Active GR can regulate gene expression of approximately 10% of the human genome [8]. Active GR is involved in development and regulation of a plethora of physiological processes including, but not limited to: inflammation, blood pressure, sensitivity to catecholamines, neuronal and glial cell activity, brain, breast, and bone development, homeostasis of body temperature, as well as carbohydrate, protein, and lipid metabolism [8]. Moreover, GCs can affect cellular processes including cell division, survival, apoptosis, migration and invasion [9, 10]; critical processes implicated in progression to a metastatic stage of cancer.

Given the heavy dependency on Dex during chemotherapy treatment, and the vast cellular processes affected by this potent steroid, we and others have examined the effects of Dex on BC cell biology. Dex has demonstrated effects on overriding cell death processes initiated by chemotherapy drugs in several solid cancers including BC [11–13]. Recently, Obradović et al. and Perez Kirkvliet et al. independently determined that GCs were able to promote BC metastasis, both in mouse PDX models and MDA-MB-231 transplants, as well as with *in vitro* trans-well assays [14, 15]. Although, Tonsing-Carter et al. showed Dex effects on ER+ cell proliferation [16], whether different receptor subtypes of BC are differentially affected by Dex has not been thoroughly investigated.

In this work we demonstrate that GR levels correlated to BC subtypes with highest expression found in the TNBCs and relatively low expression found in luminal BC cell lines. Treatment of BC cells with Dex increased overall cell numbers, invasiveness, and migratory capacity, compared to non-treated cells, and TNBCs demonstrated the most pronounced phenotypes in response to Dex. This pre-clinical work supports the growing body of data stressing the need to study the implications of Dex on BC treatment in a clinical setting.

## Materials and methods

### Cell culture

Cell lines MCF7 (HTB22; ATCC); MDA-MB-231 (HTB26; ATCC), Hs578t (HTB126; ATCC), and MDA-MB-468 (HTB132; ATCC) were cultured in Dulbecco’s Modified Eagle’s Medium (DMEM; Sigma). T47D cells (HTB-133; ATCC) were cultured in RPMI-1640 Medium (Sigma) with 0.2 units/ml of insulin (Sigma). SK-BR-3 cells (HTB-30; ATCC) were cultured in McCoy’s 5A Medium (ATCC). All cells were supplemented with 10% fetal bovine serum (FBS; Sigma) and 1% Penicillin and Streptomycin and were maintained in an atmosphere of 5% CO_2_ at 37˚C. For passaging, seeding, and quantification of cell numbers, cells were collected with 0.25% trypsin and counted using the TC10^™^ Automated Cell Counter (BioRad).

### Compounds and antibodies

The following antibodies were used at a dilution of 1:1000: actin (MAB1501R; Chemicon) and GR-α (3626–1; Epitomics). Secondary antibodies used were HRP-conjugated anti-mouse IgG (A9917; Sigma) and anti-rabbit IgG (A0545: Sigma). Dexamethasone (DN1187; BioBasic), Box 5 WNT5A antagonist (681673; Sigma), Human IgG antibody (orb27741; Biorbyt), ROR1 antibody (16540S; New England Biolabs), LGK-974(S7143; Selleckchem), Recombinant WNT5A (645-WN-010; Bio-techne).

### Immunoblotting

Samples were lysed with 0.1% NP40 buffer supplemented with Leupeptin (10 μg/ml; BioBasic), Aprotinin (10 μg/ml; Sigma), and PMSF (1 mM; BioBasic). Samples were analyzed by 10% SDS-PAGE and then transferred to a PVDF membrane. Primary antibodies were applied and incubated over-night at 4°C using dilutions specified above. Proteins were detected via treatment with Perkin-Elmer Enhanced Chemiluminscence reagent/ECL Western Gel Substrate (Perkin Elmer) and quantified using FlourChem HD2 software (AlphaInnotech; Perkin Elmer).

### Apoptotic assays

Caspase 3/7-glo assay (Promega) was used to measure the apoptotic state of treated cells. 24 h post-treatment cells were collected via trypsinization and lysed. 50 μl of Caspase-Glo® 3/7 reagent was added in each well of a white-walled 96-well plate containing 50 μl of lysis buffer as blank, negative control cell lysates, or treated cell lysates with the final concentration of 1 μg/μl. Contents were gently mixed in the wells using a plate shaker at 300–500 rpm for 30 sec. Cell lysates were incubated at room temperature for 30 min and the luminescence of each sample was measured using Wallac Victor 1420 plate reader.

### Migration and invasion assay

For migration, cells were seeded (1 x 10^5^) in 500 μL of serum-free media in 8.0μm Falcon Cell Culture Inserts (Becton-Dickinson) in the wells of a 12 well cell culture plate with 1 ml of complete media (serum-free control). Cells were treated with either ethanol (vehicle control) or different concentrations of Dex and incubated for 24 h. Following treatment, the inserts were carefully removed, cells that did not migrate through the pores and therefore remained on the upper side of the filter membrane were gently separated, and the migrated cells were quickly stained with 400 μL of 1% Crystal Violet in 2% ethanol for 10 min. The inserts were then submerged in water to remove excess Crystal Violet and air-dried. Different views of the cells attached to the membrane were imaged using a Leica inverted fluorescence microscope. The crystal violet was then released with extraction buffer, containing 10% acetic acid, and the absorption of the samples was measured at 590 nm using a Wallac Victor 1420 plate reader. For invasion assay, prior to seeding, cell culture inserts were coated with 100 μl of Cultrex® Reduced Growth Factor Basement Membrane Extract (Trevigen), diluted to 5 mg/ml, for 4 h at 37°C to gel. Remaining steps were the same as the migration assay. For the WNT5A inhibitor assay and ROR1 antibody assay, the cells were incubated with the inhibitor or antibody for 1 h prior to seeding and then the migration assay was performed as before.

### Animal care and handling

All experimental protocols and handling of Wild-type Zebrafish (*Danio rerio)* were in compliance with and approved by the University of Windsor’s Animal Care Committee (ACC) as stated in the Animal Utilization Protocol Project (AUPP#19–03) which follow regulations and standard protocols of the Canada Council on Animal Care (CCAC). Adult fish were kept at 28.5°C and bred according to protocols available in the Zebrafish Book [17] and approved by the University of Windsor’s ACC in AUPP#19–03. All applicable international, national, and/or institutional guidelines for the care and use of animals were followed.

### Implantation procedure, treatment, and imaging

Zebrafish eggs were collected after fertilization and kept in E3 embryo media (5 mM NaCl, 0.17 mM KCl, 0.33 mM CaCl_2_, 0.33 mM MgSO_4_, 10^−5^% Methylene Blue) at 28°C in an incubator until ready to inject. Before injection 500,000 MDA-MB-231 cells in 500 μl of serum-free media were labeled with 10 μL of DiI (red) (Vybrant; Invitrogen) at 37°C for 20 min. MCF7 cells were labelled with the same procedure using DiO (green) (Vybrant; Invitrogen). Cells were washed with 1ml of serum free media twice and resuspended in 50 ul of serum free media, kept at 37°C for 20 min, and placed on ice until ready to inject. Before injection both labelled cell lines were mixed at equal proportions in an Eppendorf tube. 48 h post-fertilization (hpf) the embryos were dechorionated with fine tip forceps and anesthetised with 0.168 mg/ml of Tricaine (Sigma). 50–100 of each labeled cell line/9.2 nl were loaded into glass capillary needles and injected into the yolk sac of each embryo using a Nanoject II (Fisher Scientific). After injection, embryos were placed in E3 embryo media and 1 h post-implantation (hpi) were examined using a Leica fluorescence stereomicroscope to exclude any embryo with cells outside of the implantation area. Following injection, zebrafish were transferred to 96-well plates, with one zebrafish per well. Dex was diluted to a final concentration of either 10 μM or 100 μM in E3 embryo media and added to each well of the treatment fish. DMSO was added to the control fish. 24 hpi and 24 h post treatment (hpt) the fish were anesthetized with 0.168 mg/ml Tricaine in a 96-well plate, with one embryo per well. The embryos were imaged using a Leica fluorescence microscope. This was repeated at 48 hpi and 96 hpi with fresh Dex being added at 24 hpi and 48 hpi.

All image analysis was completed using ImageJ software and was adapted from a previously described method for animal bio-imaging assays [18]. The image for each embryo was imported into ImageJ, aligned to the same orientation, and cropped to the same size. The images were converted to a 32-bit gray-scale and the threshold was adjusted to eliminate background pixels. The injection sites were chosen as the midpoint of the yolk sacs. Using the measure function, the exact coordinates for the injection site were measured. The Analyze Particle tool was then used to record the coordinates of each labeled cell foci within the entire embryo. The coordinates of each tumour foci were corrected to the injection site coordinates using the formula: (X_foci_-X_origin_,Y_foci_-Y_origin_)_._ For each corrected foci coordinate the distance travelled from the injection site was calculated using the formula: √(X_corrected_^2^+Y_corrected_^2^). The cumulative distance (CD) of all foci was calculated per embryo and averaged within an experimental group to determine mean CD. Each embryo was scored as having either cells metastasized to the tail or no metastasis and the percentage of fish with metastases was calculated.

### qRT-PCR analysis

MCF7 and MDA-MB-231 cells were collected after Dex treatment and RNA extracted using RNeasy Plus Mini Kit (Qiagen; 74134). Reverse Transcription of 1ug of RNA was performed using the established QuantaBio qScript cDNA Supermix protocol. Relative RNA expression was measured using *GAPDH* as an endogenous control. The following sequences were used: *MMP9* [19] 5′-ACGCACGACGTCTTCCAGTA-3′ and 5′-CCACCTGGTTCAACTCACTCC-3′; *TGFβ1* [20] 5′-TCGCCAGAGTGGTTATCTT-3′ and 5′-TAGTGAACCCGTTGATGTCC-3′; *WNT5A* [21] 5′- TAAGCCCAGGAGTTGCTTTG- –3′ and 5′- GCAGAGAGGCTGTGCTCCTA-3′; *IL1B* [22] 5′- CATGGCCACAACAACTGACG-3′ and 5′- AGCCATGGCAGAAGTACCTG -3′; *IL6* [23] 5′- GAGATGCCGTCGAGGATGTA-3′ and 5′- CTTCGGTCCAGTTGCCTTCTC -3′.

### Statistical analysis

Student *t* test or one-way Anova were employed using Statistica and GraphPad software. For percent metastasis the two-proportion Z test was performed. All other results are expressed as mean ± SEM and differences were considered significant at p values of <0.05.

## Results

### TNBCs express higher levels of GR-α

**Fig 1:**
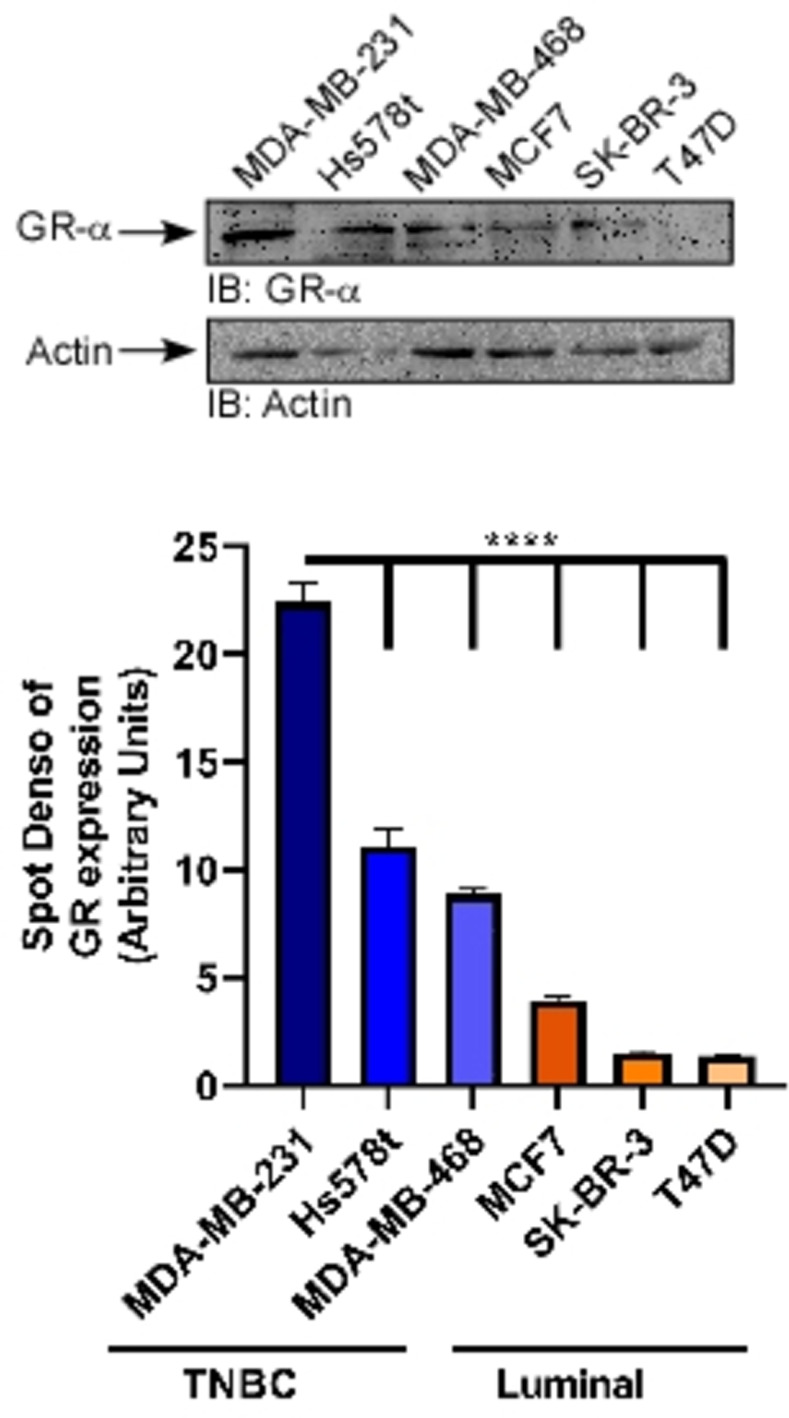
Relative expression of GR-α in triple negative and luminal BC cell lines. TNBC cell lines MDA-MB-231, Hs578t, and MDA-MB-468 and luminal BC cell lines MCF7, SK-BR-3 and T47D were subjected to western blotting. Endogenous GR-α levels were measured by immune-blotting. Densitometry analysis of three separate experiments, indicating GR-α protein levels normalized to actin, (lower panel) is represented as mean ± SEM. ****p ≤ 0.0001. Original western blots presented are available in S1 Fig.

We assessed the relative levels of endogenous GR-α across a panel of BC cell lines. Three TNBC (MDA-MB-231, Hs578t, MDA-MB-468), and three luminal subtype (MCF7, SK-BR-3, T47D) cell lines were used (Fig 1). The highest levels of GR protein were expressed in TNBC cell lines with the highly aggressive MDA-MB-231 cells displaying the highest levels, followed by the Hs578t cells and the MDA-MB-468 cells, respectively. Expression was significantly reduced in the luminal cell lines with MCF7s demonstrating the highest expression of the luminal subtype.

### Dex increases cell numbers of BC cells *in vitro*

**Fig 2:**
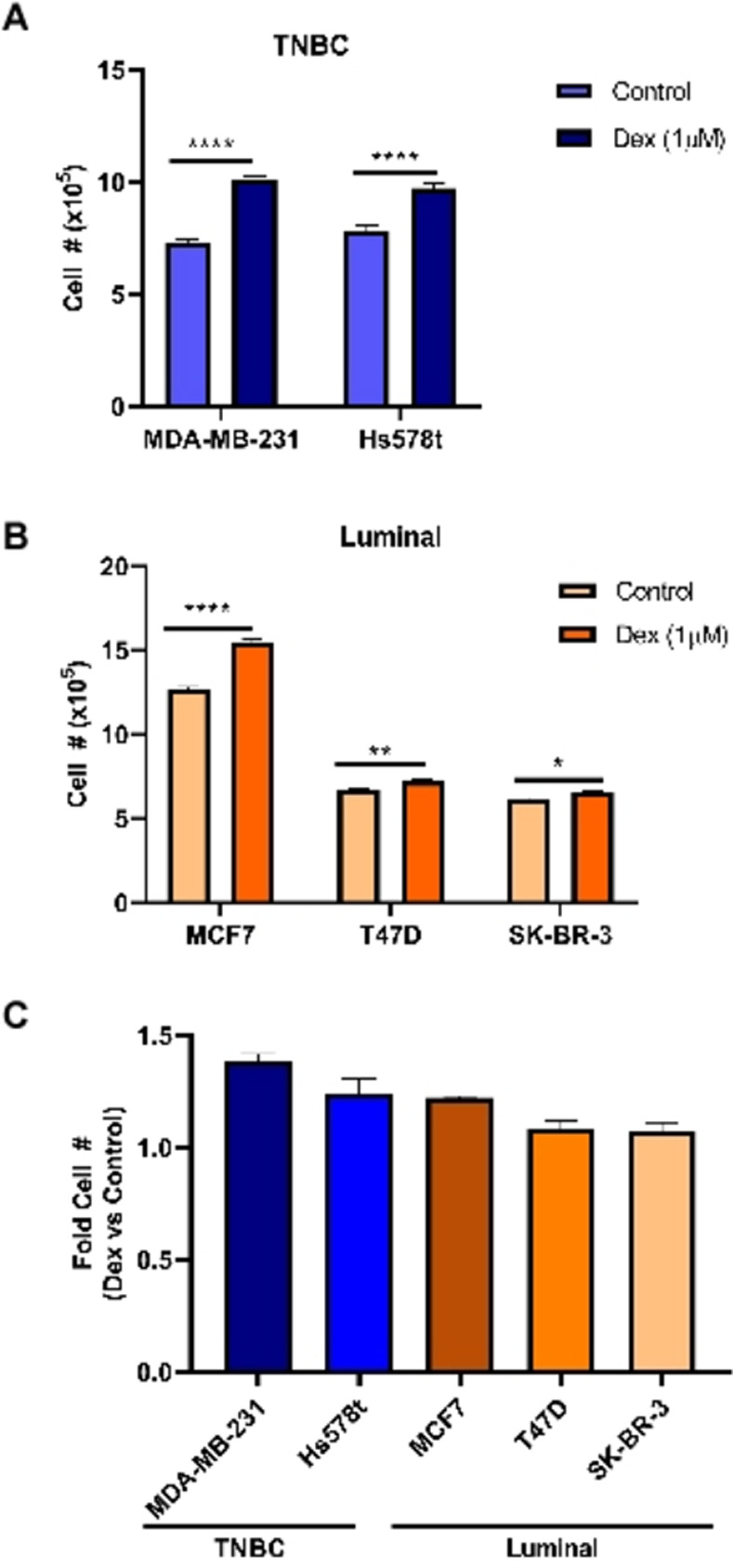
Impact of Dex on total cell number 24 h post-treatment. **a** TNBC cell lines MDA-MB-231 and Hs578t and **b** luminal BC cell lines MCF7, SK-BR-3 and T47D were treated with vehicle (control) or Dex for 24 h. Cells were collected and counted for total cell number. **c** Graphic representation of the fold-change in Dex-treated cells relative to vehicle of each respective cell line. Graphs show the mean value of at least three experiments, each performed in triplicate, upon which statistical analysis was performed. *p ≤ 0.05, **p ≤ 0.01, ****p ≤ 0.0001.

After treatment with Dex, the TNBC cell lines, MDA-MB-231 and Hs578t, showed the greatest increase in total cell number at 38% and 24% compared to non-treated cells (Fig 2A and 2C). The highest cell number amongst the luminal BC cell lines correlated with GR-α expression in the MCF7s with 22% higher count in Dex-treated compared to non-treated cells (Fig 2B and 2C). The SK-BR-3 and T47D cell lines displayed the smallest difference in cell number between treated and non-treated at 8% and 7% difference, respectively (Fig 2B and 2C).

### Dex increases migration of TNBC cell lines *in vitro*

**Fig 3:**
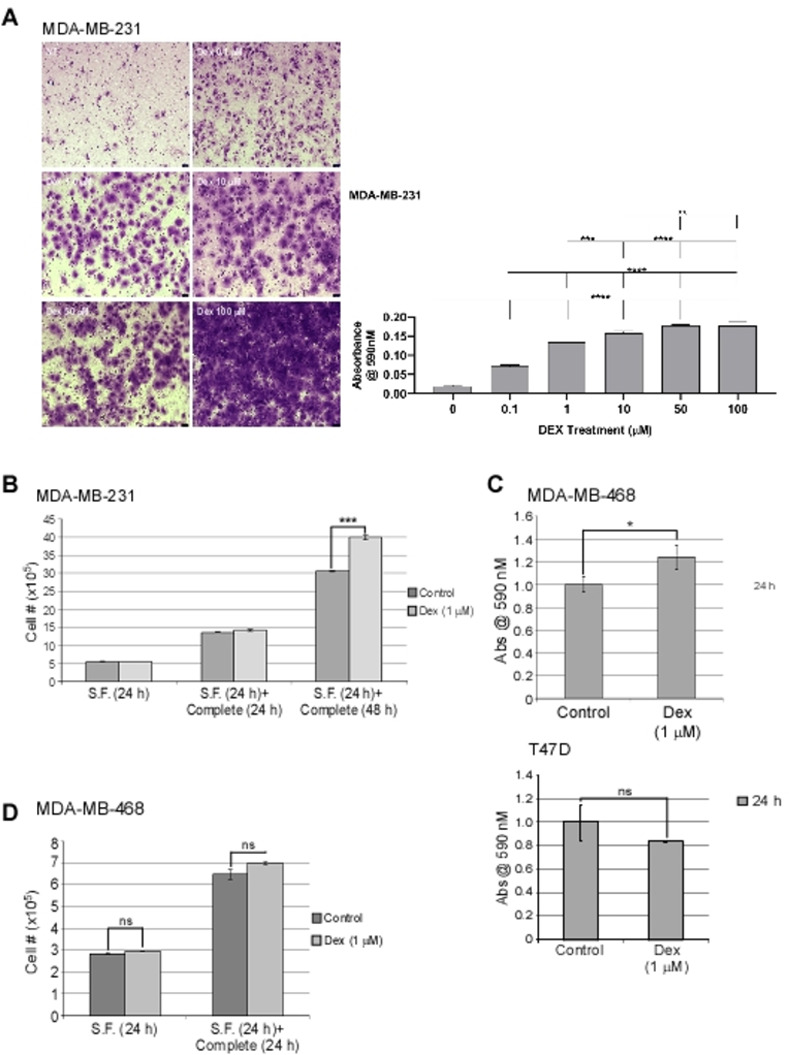
Impact of Dex on migration of triple negative and luminal BC cell lines *in vitro*. **a** TNBC cell lines MDA-MB-231 were treated with vehicle (control) or with Dex and migration of the cells was visualized by microscopic images (left panels). Graph representing quantification of migrated cells (right panels). **b** Graph representing Cell # of MDA-MB-231 cells after treatment with Dex in Serum Free or complete media. **c** Graph representing quantification of migrated cells with MDA-MB-468 and T47D cell lines **d** graph representing Cell # of MDA-MB-468 cells after treatment with Dex in Serum Free or complete media. n = 3. Graphs represented as mean ± SEM.*p<0.05, **p<0.01, ***p ≤ 0.001, ****p<0.0001.

TNBC and ER+ cells were treated with vehicle or a dose range of Dex in serum-free media and seeded into a transwell assay where a chamber containing complete medium was separated by a membrane. Cells capable of migrating across the membrane were measured. A significant increase in migration was observed compared to control cells in the MDA-MB-231 and MDA-MB-458 cell line (Fig 3A and 3C). The luminal BC cell line T47D and MCF7 cell line did not display an increase in migration following Dex treatment (Figs 3C and S2).

To ensure that increases in cell number are not influenced by Dex-induced proliferation or enhanced survival, cells were cultured in combinations of serum-free conditions for 24 h, or serum free for 24 h followed by the addition of supplements (to make the medium ‘complete medium’) for 24 or 48 h (Fig 3B and 3D). There was no change in cell number between Dex treated and non-treated cells in serum-free media conditions over a 24 h period, or in complete media for 24 h (Fig 3B and 3D). It is notable that when MDA-MB-231 cells were left for an additional 24 h (total time in complete media = 48 h), a significant difference in cell number was observed for Dex-treated cells suggesting a Dex-mediated proliferative or pro-survival advantage at the site of migration (Fig 3B). To measure apoptosis, we analyzed caspase 3 and 7 activity under the same conditions using the TNBC cell lines (MDA-MB-468 and Hs578t) and the luminal (SK-BR-3 and T47D) cell lines. All four cell lines showed decreases in caspase 3 and 7 activity (S2A and S2B Fig). The cell lines expressing the highest levels of GR-α also demonstrated greater sensitivity to Dex-mediated inhibition of the caspases. The Hs578t cells showed 60.7% decrease in caspase 3 and 7 activity. The luminal cell lines SK-BR-3 and T47D displayed 30.9% and 34.4%, respectively.

### Dex increases invasiveness of BC cell lines *in vitro*

**Fig 4:**
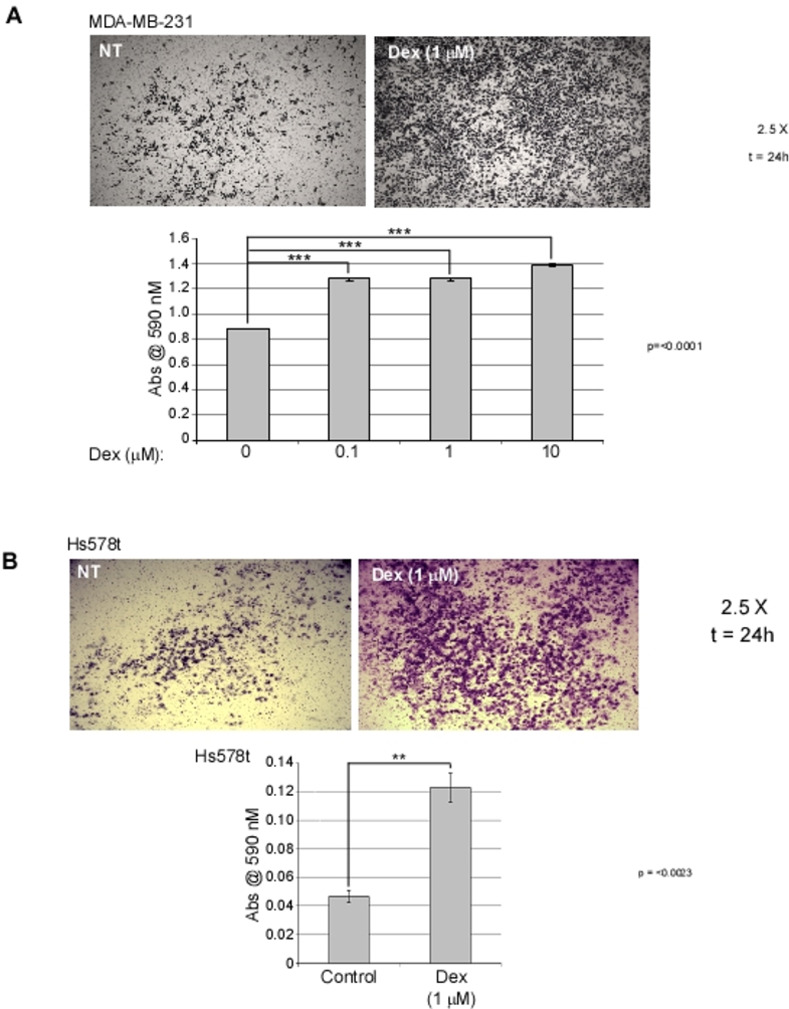
Dex differentially affects invasion of ER- and ER+ cells *in vitro*. Microscopy images and graphs representing invasion of **a** MDA-MB-231 and **b** Hs578t cells with and without Dex treatment. Date represents n = 3 and are presented as the means ± SEM. **p ≤ 0.01, ***p ≤ 0.001.

To assess the effect of Dex on BC cell lines *in vitro*, cells were treated with vehicle (control) or with increasing concentrations (0.1–10 μM) of Dex in the transwell system where the membrane was coated with Cultrex BME. We observed an increase in invasiveness with increases in Dex concentration with both MDA-MB-231 and Hs578t cells (Fig 4).

### Dex enhances metastatic properties of BC cell lines *in vivo*

**Fig 5:**
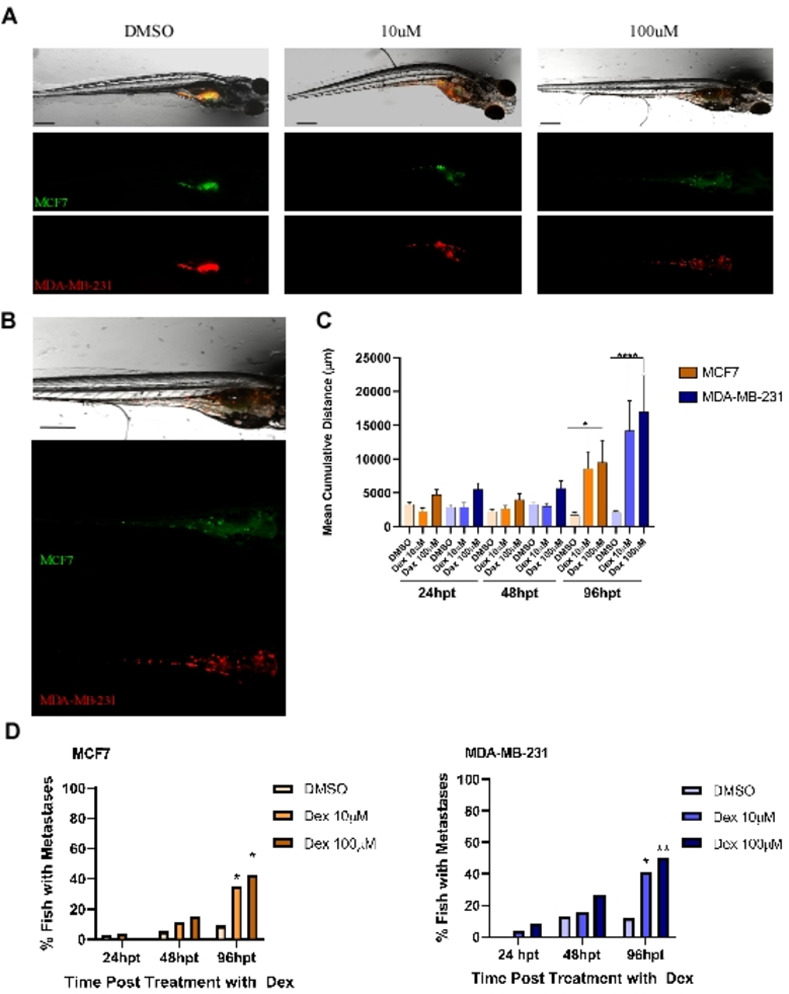
Metastasis of BC cells in zebrafish xenotransplants. **a** Representative brightfield and fluorescence images of embryos 96 h after treatment with either DMSO, 10 μM or 100 μM Dex. Images were taken at 40x magnification. MCF7 cells are shown in green and MDA-MB-231 cells in red. **b** Enlarged images of representative fish from 96 h after treatment with 100μM Dex. White arrows show the cell foci that have migrated outside of the yolk sac. **c** Mean cumulative distance of the tumour foci from the yolk sac in MCF7 and MDA-MB-231 cells. **d** Percentage of fish that had metastases outside of the yolk sac. n = 14–33 fish. Scale bar = 300 μm. *p<0.05, **p<0.01.

To examine the effect of Dex on BC cell behavior *in vivo*, MDA-MB-231 and MCF7 cells were used in an *in vivo* zebrafish xenograft assay. Both cell lines were injected together into the yolk sac of fish and cells were able to invade outside of the yolk and form quantifiable metastatic tumour foci. 96 h post-injection (and treatment) approximately 10% of fish had measurable tumour burden at distant sites (Fig 5A–5D). Interestingly, within both cell lines, Dex treatment increased the mean cumulative distance of the foci from the yolk sac (Fig 5C) and at the 96 h time point the number of fish with measurable metastases was 4-fold higher than the DMSO control; however, between cell lines there was a 1.5-fold difference in cumulative distance travelled and metastases was only modestly increased in MDA-MB-231 cells at the 96 h time point as compared to MCF7 cells (Fig 5D). While co-injection allows direct comparison of the cell behaviour within each fish, it is also notable that cross-talk and cell-cell interactions may have increased migratory potential of the ER+ population *in vivo*. However, consistent with differences seen in *in vitro* assays, MDA-MB-231 cells treated with Dex travelled 6-fold further from injection site compared to DMSO treated cells whereas the MCF7 Dex treated cells travelled 3.6-fold further than DMSO treated cells. MDA-MB-231 cells also had ~5% increase in final tumour burden over the luminal MCF7 line.

### Dex causes changes in inflammatory gene expression uniquely in TNBC cells

**Fig 6:**
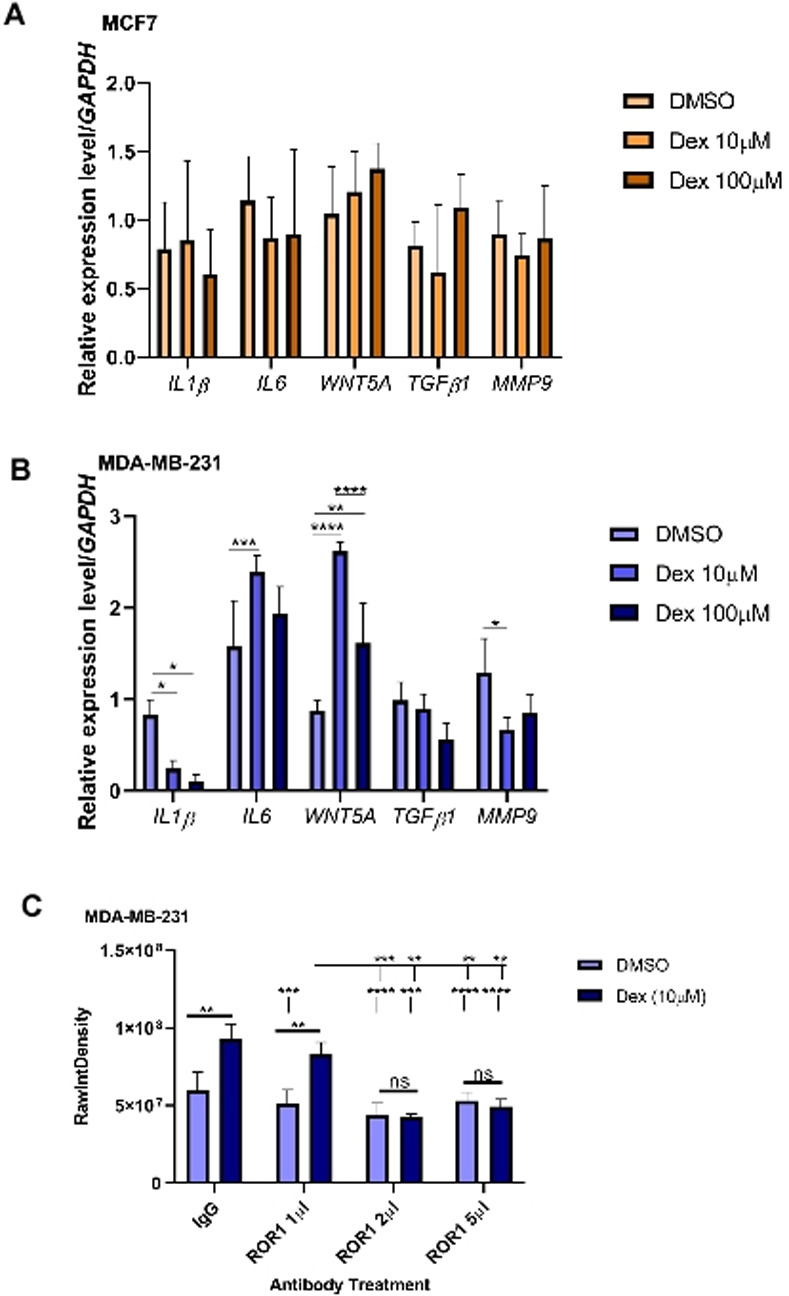
WNT5A activation is necessary for Dex mediated migration in select BC cell lines. Relative gene expression of **a** MCF7 or **b** MDA-MB-231 cells after 96 h Dex treatment at indicated concentrations *in vitro*. **c** Graph representing the relative migration of MDA-MB-231 cells in the presence and absence of Dex treatment with and without ROR1 blocking antibody. Graphs represent mean ± SEM. n = 3; *p<0.05, **p<0.01, ***p<0.001, ****p<0.00001.

Metastases from mice implanted with TNBC cells and then treated with Dex showed protein enrichment of inflammatory pathways and activation of ROR1 [14]. It has also been shown in an independent study that ROR1 activation by WNT5A promotes breast cancer migration and metastasis [24]. Here, we treated TNBC and ER+ cells in culture with Dex and looked at expression of inflammatory genes to determine if Dex directly causes upregulation of inflammatory pathways. The ER+ MCF7 cells had no difference in inflammatory gene expression after Dex treatment (Fig 6A). After treatment, the TNBC MDA-MB-231 had significant downregulation of *IL1B*, with both concentrations, and *MMP9* after 10μM. Interestingly, Dex significantly increased *WNT5A* expression with both concentrations and increased *IL6* expression after the 10μM treatment (Fig 6B). In two other TNBC cell lines (Hs578t, MDA-MB-468) *WNT5A* gene expression was not as prominent after Dex treatment, albeit Hs578t did increase gene expression (S3A and S3B Fig).

To determine if WNT5A was contributing to Dex mediated migration, TNBC cells were treated with a ROR1 blocking antibody prior to the transwell migration assay to block the WNT5A/ROR1 interaction. Migration was significantly decreased with increasing concentrations of the ROR1 antibody in the MDA-MB-231 cells. In the MDA-MB-468 cells, Dex mediated migration was initially ablated but higher concentrations of ROR1 ab started to show a reversion back to increased Dex migration and an increase in migration within the DMSO treated cells. Further investigation is needed to determine if an alternate pathway is being activated to compensate for the loss of ROR1 function (Figs 6C and S4). However, migration of cells (in both TNBC and ER+) was unchanged when a WNT5A inhibitor was used to block the WNT5A/Frizzled-5 interaction (S5 Fig).

**Fig 7:**
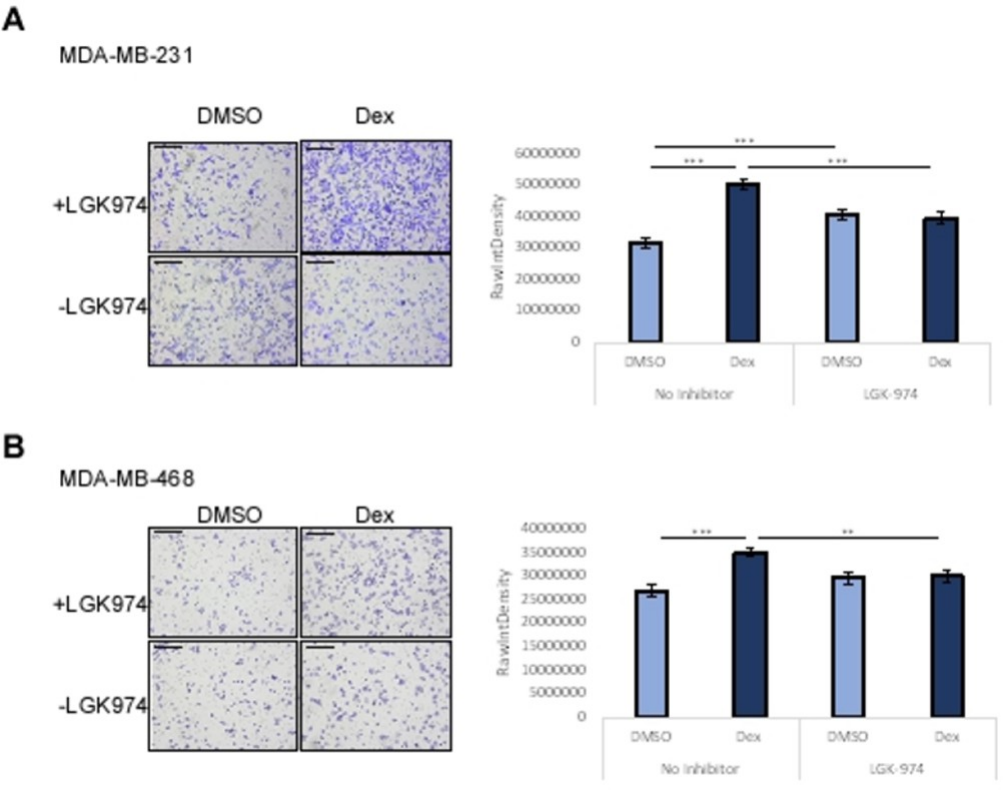
LGK-974 can prevent Dex mediated migration. **a** MDA-MB-231 and **b** MDA-MB-468 cells were treated with 10μM Dex in the presence and absence of 1μM LGK-974. Migration measured 24 hours post treatment. Error bars reflect standard error (SE), Student’s T test *p < 0.05, **p < 0.01, ***p < 0.001.

To confirm the dependence of Wnt ligands to activate the Ror1 pathway after Dex treatment, we treated TNBC cells with a Porcupine inhibitor, in both DMSO and Dex treated cells, to prevent Wnt secretion. In both MDA-MB-231 and MDA-MB-468 cells there was a complete reversion of migration back to levels seen in DMSO treated cells (Fig 7A and 7B). However, the Hs578t Dex treated cells retained their enhanced migratory abilities after the addition of the LGK974 inhibitor (S6 Fig).

### Sustained WNT5A expression is not sufficient alone for breast cancer migration

To test whether WNT5A alone was sufficient to induce migration, recombinant WNT5A was added to all 3 TNBC cell lines for 24 hours in the presence or absence of Dex. In all cases Dex continued to increase migration, however cells stimulated with WNT5A did not increase migration alone and in most cases decreased migration (S7 Fig).

## Discussion

Metastatic BC cells present several key characteristics, namely 1) the ability to survive by eluding apoptosis and continuing to grow and proliferate at the primary tumour site; 2) the ability to invade through surrounding tissue and 3) the ability to migrate in circulation or within neighboring tissue [25]. These characteristics can be augmented by drug treatment. Thus, the effect of clinical therapies and adjuvant drugs on BC cell characteristics is of clinical significance to the progression of metastasis.

In chemotherapy regimens, the steroid Dex is administered in advance of chemotherapy to alleviate allergic and hypersensitivity reactions as well as nausea and vomiting in patients [26, 27]. This work demonstrates that administration of Dex increases cell number, migratory capacity and invasiveness within 24 h post-treatment relative to vehicle-treated control cells *in vitro* (Figs 2–4). Previous reports claim that Dex increases cell proliferation in solid cancer cells [10, 28]. These previous studies used only cell viability assays and no counts were performed. While we did perform cell counts, neither our data, nor those reports, excludes the possibility that increased numbers in Dex-treated cells versus non-treated cells is due to increased survival and not proliferation. At most we show that Dex permits cells to continue dividing better than those that did not receive Dex (Fig 2A and 2B). Thus, differences in cell number compared to control could be attributable to enhanced cell survival with Dex. Zheng *et al*. also report that their claims of increased proliferation were not corroborated by increases in the proliferative markers of cyclins and CDKs [10]. Pang *et al*. also qualify data concerning Dex-mediated increases in tumour size that did not show increased expression of Ki-67 as most likely being due to an increase in survival [29]. Further analysis with BrdU incorporation or Ki-67 expression could give insight into this matter of concern.

We also demonstrate that Dex-treated cells had increased motility as evident in migration and invasion assays (Figs 3 and 4). A search of the literature shows several reports on Dex as reducing migration and invasion [9, 10, 30, 31]. While these reports are also in other tissue types, we sought to determine whether there were alternate explanations for our data. One possibility is that the Dex-treated cells were surviving and/or proliferating faster, producing more cells on the pre-migration and pre-invasion side of the chambers compared to control chambers. Thus, even if equal percentages of cells migrated or invaded thereafter, the Dex-treated chambers would have more cells to migrate or invade and a selective advantage over control chambers. Another putative explanation is that while Dex would confer no advantage in cell number pre-migration or invasion, Dex treated cells would be ‘primed’ for increased proliferation or survival compared to control cells once they reached the complete media post-migration or post-invasion. In this model equal numbers of cells exist in both the control and Dex-treated cell chambers pre-migration or invasion and equal numbers of cell migrate or invade. Once these cells reach complete media, however, the Dex treated cells proliferate sooner and thus account for differences in the assay. We demonstrate that neither of these explanations can account for the observed data in the conditions and brief timeframe that the experiment takes place (Fig 3B and 3D). Given longer periods of time, however, we show that the latter explanation could be true and further supports our earlier report that Dex enhances survival and/or proliferation in BC cells (Fig 3B).

Our *in vivo* data supports the role of Dex in enhancing one, if not more, characteristics of metastatic BC cells (Fig 5). Zheng *et al*. attributed increases in tumour mass in mice xenografts of Dex treated mice to increased survival of tumour cells resulting in larger tumours [10]. It is possible that increased survival of injected cells *in vivo* contributes to final tumour burden. However, our data also shows a very significant increase in the distance travelled with Dex treatment and our *in vitro* assays support the ability of Dex to modulate cell motility.

It has been reported that Dex treatment causes upregulation of pathways involved in inflammation and metastasis [14], specifically *WNT5A* and *ROR1* expression but it was only shown in TNBC. We wanted to determine if Dex would have the same effect in ER+ MCF7 cells. Interestingly, the MCF7 cells showed no significant difference in relative expression of any of the inflammatory genes examined (Fig 6A). As expected, in the MDA-MB-231 cells, Dex caused a significant increase in *WNT5A* expression (Fig 6B). *IL1B* expression was significantly downregulated whereas *IL6* expression was upregulated which means Dex is only activating certain inflammatory pathways and though MCF7 cells can metastasize *in vivo* it might be through another mechanism than TNBC cells. Our data supports that Dex mediated migration may utilize the WNT5A/ROR1 interaction and was independent of the WNT5A/Frizzled-5 interaction (Figs 6C and S2). Some previous reports have shown a negative correlation with WNT5A signaling and breast cancer cell migration through the addition of recombinant WNT5A(rWNT5A) to MDA-MB-231 cells [32]. We also saw this effect with larger concentrations of rWNT5A when cells were treated for 24hrs or more (S7 Fig) but our results show the efficacy of the LGK974 inhibitor and the Ror1 blocking antibody in reversing Dex mediated migration, suggesting that WNT5A may be a component of Dex mediated effects in select BC cell lines. Conflicting results with the Hs578t cell line demonstrating increasing migration with increased Ror1 blocking antibody may be due to the use of alternate signaling pathways in this particular cell line that may become upregulated in the presence WNT5A pathway inhibition. Inhibition of migration seen with rWNT5A could be due to cell toxicity to the sustained exposure to WNT5A once the cells have migrated as previous data shows short exposure to WNT5A does increase migration of MDA-MB-231 cells [33]. While this work does not point to a definitive role for WNT5A signaling in Dex mediated migration, it does highlight the importance of characterization of Dex mediated signaling in multiple subtypes and cell lines as critical differences are likely to be uncovered. Future characterization of the potential involvement of ROR2, and the balance of other Wnt ligands is necessary to understand cell specific variability in this response. It would also be important to address whether Wnt signaling could be a clinical target to permit optimal use of Dex in TNBC patients.

Overall, this work adds to the growing body of data which supports that Dex can affect multiple parameters supporting metastatic events *in vivo*. Given the ubiquitous use of Dex in treating BC patients with the most severe forms of BC, further research into elucidating Dex-mediated effects on BC cell behavior is warranted.

## Supporting information

S1 Fig Original western blot and overlay. (JPG)

S2 Fig Dex decreases apoptosis. (JPG)

S3 Fig Differential gene expression following dexamethasone treatment. (JPG)

S4 Fig Inhibition of Ror1 blocks Dex mediated migration. (JPG)

S5 Fig Dex increases migration independently of WNT5A/Frizzled-5 interaction. (JPG)

S6 Fig LGK-974 does not affect Dex mediated migration in Hs578t cells. (JPG)

S7 Fig Recombinant Wnt5a reduces migration. (JPG)

S1 Raw images (PDF)
