# Supplementary figures and images for: Frequently used antiemetic agent dexamethasone enhances the metastatic behaviour of select breast cancer cells
Source: PLoS One. 2022 Sep 15;17(9):e0274675. doi: 10.1371/journal.pone.0274675 (PMC9477352; doi:10.1371/journal.pone.0274675)

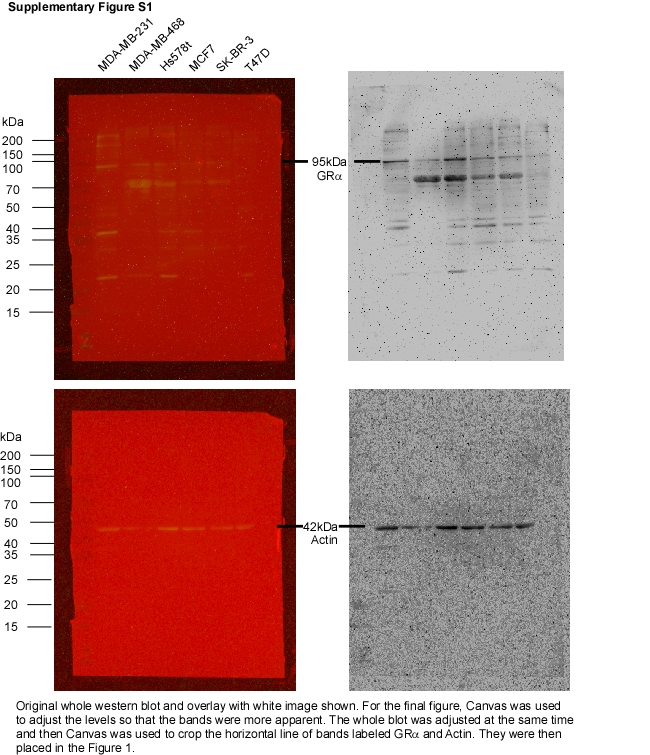

Supplement: S1 Fig — (JPG) [file pone.0274675.s001.jpg]

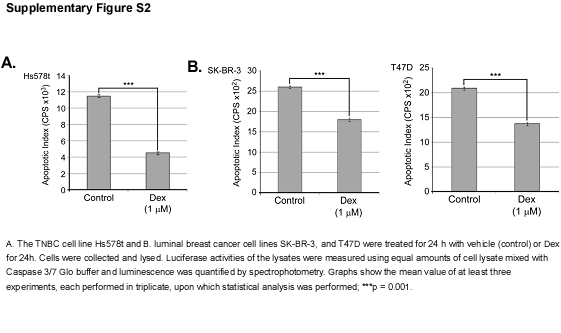

Supplement: S2 Fig — (JPG) [file pone.0274675.s002.jpg]

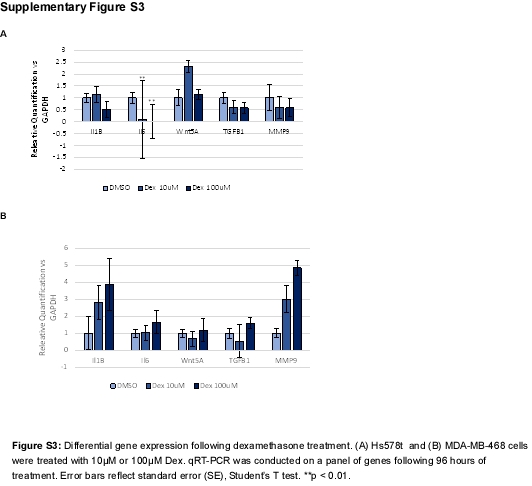

Supplement: S3 Fig — (JPG) [file pone.0274675.s003.jpg]

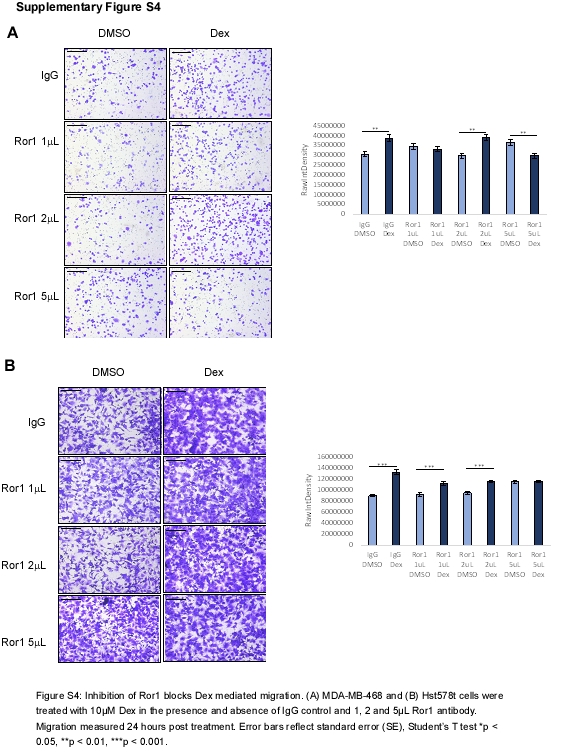

Supplement: S4 Fig — (JPG) [file pone.0274675.s004.jpg]

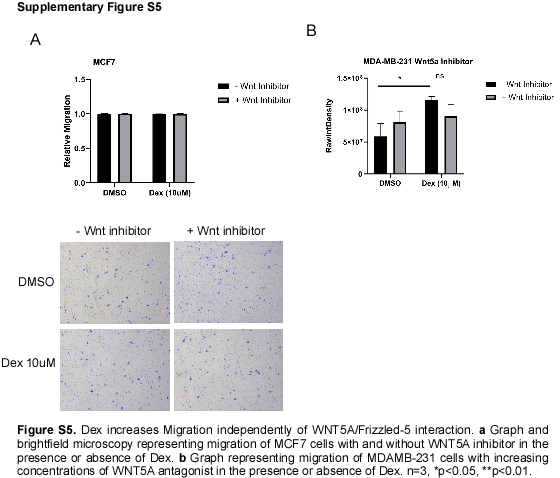

Supplement: S5 Fig — (JPG) [file pone.0274675.s005.jpg]

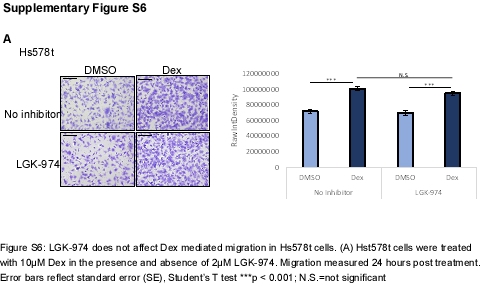

Supplement: S6 Fig — (JPG) [file pone.0274675.s006.jpg]

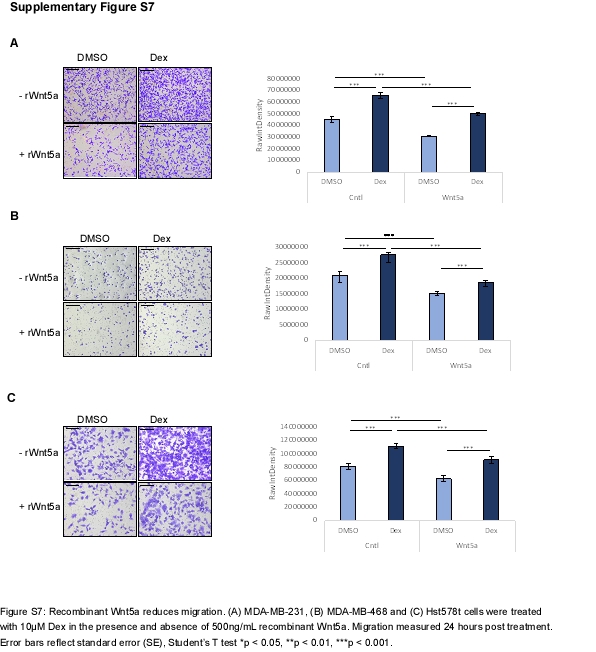

Supplement: S7 Fig — (JPG) [file pone.0274675.s007.jpg]
