# Supplementary material for: Frequently used antiemetic agent dexamethasone enhances the metastatic behaviour of select breast cancer cells
Source: PLoS One. 2022 Sep 15;17(9):e0274675. doi: 10.1371/journal.pone.0274675 (PMC9477352; doi:10.1371/journal.pone.0274675)
Supplement: S1 Raw images — (PDF) [file pone.0274675.s008.pdf]

A)

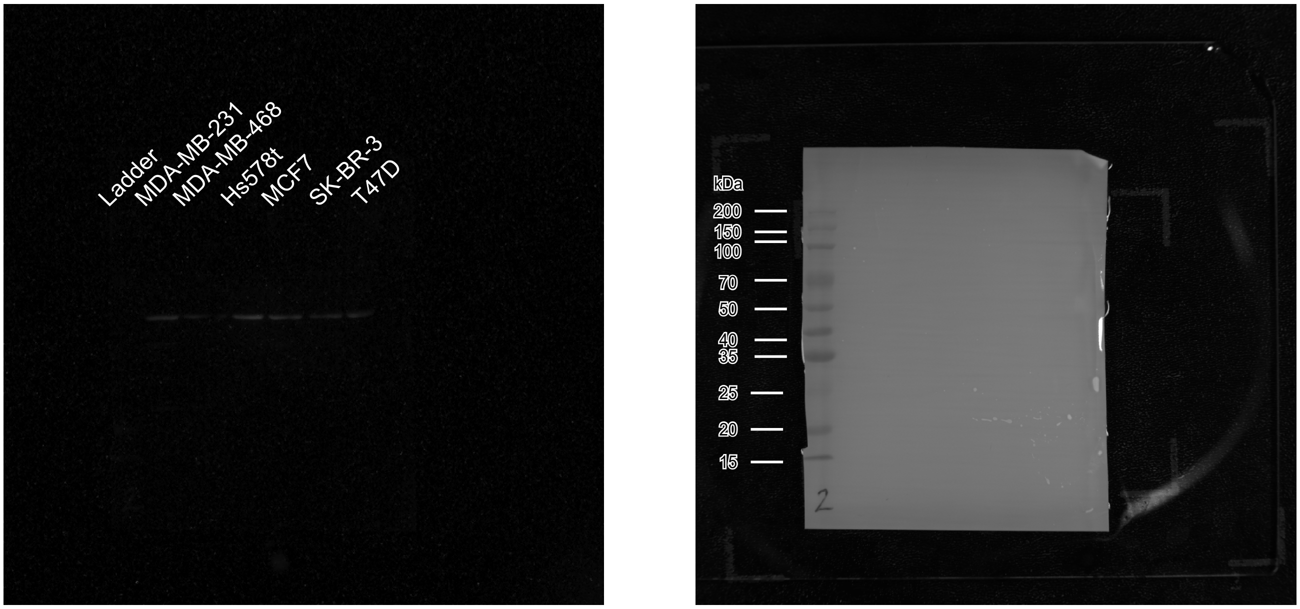

B)

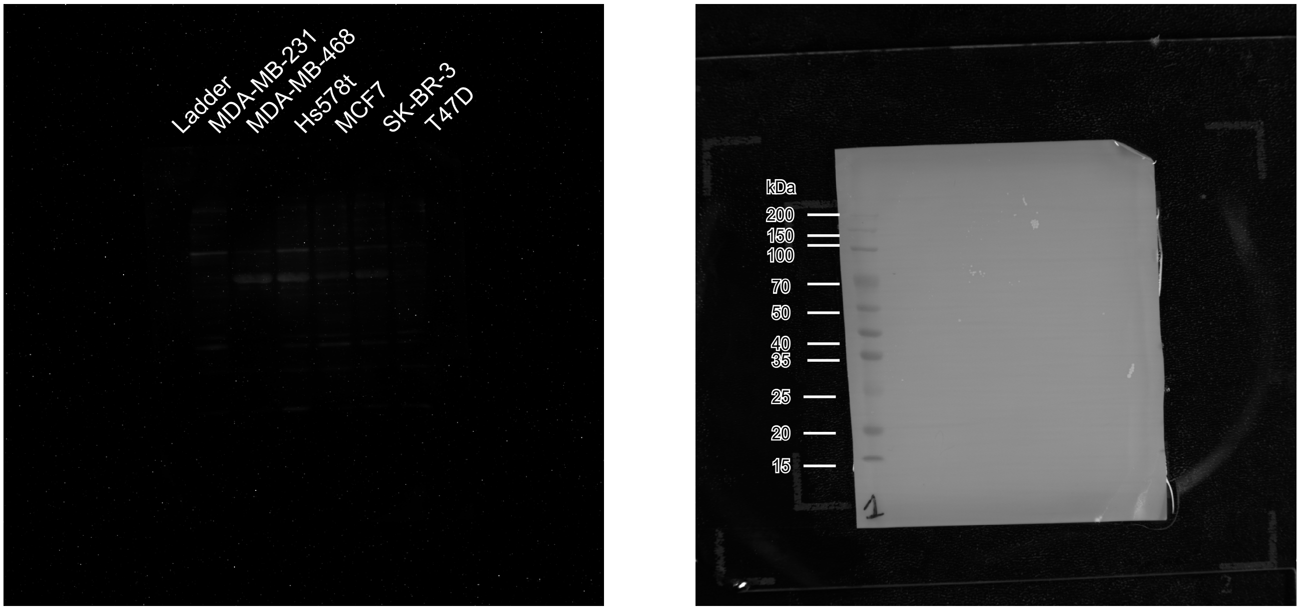

Proteins were detected via treatment with Perkin-Elmer Enhanced Chemiluminscence reagent/ECL Western Gel Substrate (Perkin Elmer) and quantified using FlourChem HD2 software (AlphaInnotech; Perkin Elmer).

A) Chemiluminescence and brightfield image of the membrane showing blot for Actin. B) Chemiluminescence and brightfield image of the membrane showing blot for Glucocorticoid Receptor. These blots were cropped and used in Figure 1 and the uncropped overlays were used in Supplemental Figure S1.
